# Drilling characteristics and properties analysis of fiber reinforced polymer composites: A comprehensive review

**DOI:** 10.1016/j.heliyon.2023.e14428

**Published:** 2023-03-13

**Authors:** Praveenkumara Jagadeesh, Sanjay Mavinkere Rangappa, Indran Suyambulingam, Suchart Siengchin, Madhu Puttegowda, Joseph Selvi Binoj, Sergey Gorbatyuk, Anish Khan, Mrityunjay Doddamani, Vincenzo Fiore, Marta María Moure Cuadrado

**Affiliations:** a Natural Composites Research Group Lab, Department of Materials and Production Engineering, The Sirindhorn International Thai-German Graduate School of Engineering (TGGS), King Mongkut’s University of Technology North Bangkok (KMUTNB), Bangkok, Thailand; b Department of Mechanical Engineering, Malnad College of Engineering, Hassan, Visvesvaraya Technological University, Belagavi, Karnataka, India; c Institute of Mechanical Engineering, Saveetha School of Engineering, Saveetha Institute of Medical and Technical Sciences, Chennai, 602105, Tamilnadu, India; d Department of Engineering of Technological Equipment in National University of Science and Technology MISIS, Moscow, Russia; e Chemistry Department, Faculty of Science, King Abdulaziz University, Jeddah, Saudi Arabia; f School of Mechanical and Materials Engineering, Indian Institute of Technology Mandi, Mandi, 175075, Himachal Pradesh, India; g Department of Engineering, Università degli Studi di Palermo, Viale delle Scienze, Palermo, Italy; h Aerospace Systems & Transport Research Group, Rey Juan Carlos University, Madrid, Spain

**Keywords:** FRP composites, Drilling, Delaminations, Thrust force, Natural fibers, CFRP, GFRP

## Abstract

Fiber-reinforced polymer (FRP) composites play a vital role in the production of structural and semi-structural components for engineering applications. The drilling process is a commonly employed machining process for FRP composites to join the FRP structural elements. Usually, the FRP composites possess a heterogeneous nature because of their multi-layered structure, hybridization, and the presence of multi-phase materials. Hence, common problems like delaminations, fuzzing, buckling, cracking, matrix and fiber burning occur during the drilling operations. These problems cause dimensional inaccuracy, poor surface finish, and tool wear and reduce the mechanical strength of the composites. The optimum drilling parameters (drill geometry, speed, feed, and depth of cut) selection for the specific materials is good to achieve effective drilling performance and better surface quality of the holes. Yet, little study has been done on how all of these factors affect the size of the drilled hole. The majority of drilling studies on FRPCs in the past have focused on how to improve the hole quality by maximizing processing conditions, and there has been little discussion on the correlation between drilling conditions, physical properties, and production techniques. This is what motivated to review the characteristics and properties analysis of FRP composites. As a consequence of this research, it is anticipated that scientists and researchers would place a greater emphasis on the drilling characteristic of the workpieces made from FRPCs than on other attributes. This review clearly presents an overview of FRP composites drilling that had progressed from 2000 to 2021. The analysis of different drilling conditions and parameters like thrust force, drill geometry, temperature, speed, and feed also includes the post-drilling analysis through delaminations, thermal damage, and surface roughness. Furthermore, the recent developments in carbon, glass, and natural fiber reinforced polymer composites are studied with both conventional and nonconventional drilling techniques. Based on the above studies, some future challenges and conclusions are drawn from this review.

## 1 Introduction

Polymer composites are the type of versatile, high-potential materials that are formed with different phases of materials, of which at least one is a matrix and the other is a polymer material. The combination of fiber reinforcement with polymer matrix gives unique physical, thermal and mechanical properties which are not possible with single material [[1], [2], [3]]. The addition of fiber reinforcement increases the stiffness, tensile strength, heat resistance, chemical resistance, conductivity, corrosion resistance, and matrix interaction of polymer matrices and composites [4,5]. Usually, the researchers were preferred to use two types of fibers as a reinforcement, they are natural and synthetic fibers. The reinforcing fibers in fiber-reinforced polymer (FRP) composites serve as a load-bearing element and have a higher strength than the matrix materials. The FRP composite performance dependent on various factors, such as the polymer matrix properties, fiber properties, fiber orientation, fiber geometry, and fiber to matrix volume ratio [[6], [7], [8]]. Their potentiality led to applicability in various sectors like the power industry, construction field, consumer goods, electrical equipment, automotive, and aerospace industries. In recent years, researchers are trying to include bio-based fibers for FRP composite in concern towards environmental aspects and renewability. Carbon fiber and glass fiber reinforced polymer composites, which are currently employed in the production of a variety of goods such pipes, tanks, turbine blades, and printed circuit boards, are the most well-known and well-established FRP composites. The glass fiber reinforced polymer (GFRP) composites are extensively popular for engineering applications in various sectors like aerospace, process industries, gas, and oil industries. These GFRP composites have a lesser relative density than steel and a higher stiffness value than aluminum material. The usage of low-density CFRP composites in product development led to advantages like lightweightness, low cost, and flexibility in designing complex structures. The carbon fibers reinforcement in polymeric materials impose electrical properties and are suitable for multifunctional parts production. Although, these are specifically capable to protect or de-icing aircraft wings from the thunder strike or storing energy [[9], [10], [11]].

The drilling process is essential to facilitate part assembly in industries. The FRP composites drilling was more complicated than the metals due to the passage of drill through alternative layers of reinforcements and matrix having unique properties. Due to the harsh, abrasive, and heterogeneous nature of the fiber components, delamination occurs frequently throughout the drilling process [12,13]. The 60% of manufactured parts using FRP composites were rejected at the assembly points due to dimensional inaccuracy and poor surface finish [14,15]. In comparison to conventional materials drilling, FRP composites drilling has so many other problems such as fiber breakage, fibers debonding, spalling, uncut fibers, fibers chipping, etc. The FRP composites drilling is critical for the riveted joints where there is a requirement of higher quality holes without affecting their residual strength [16,17]. Due to delaminations and microcracking, FRP composites struggle to meet standards including axial straightness, waviness-free interiors, and roundness. The higher stress concentration results in the decrease of residual mechanical characteristics also cause resolidification and softening of matrix materials. This phase change phenomenon results in the variation of thermal properties of fiber as compared to base material properties. In contrast to metals, plastics often have lower transition temperatures and thermal conductivity coefficients, which causes the matrix material to burn and suffer heat degradation. The rule of mixture Eq. (1) is used to compute the heat conductivity of the FRP composite [18,19].

**Fig. 1:**
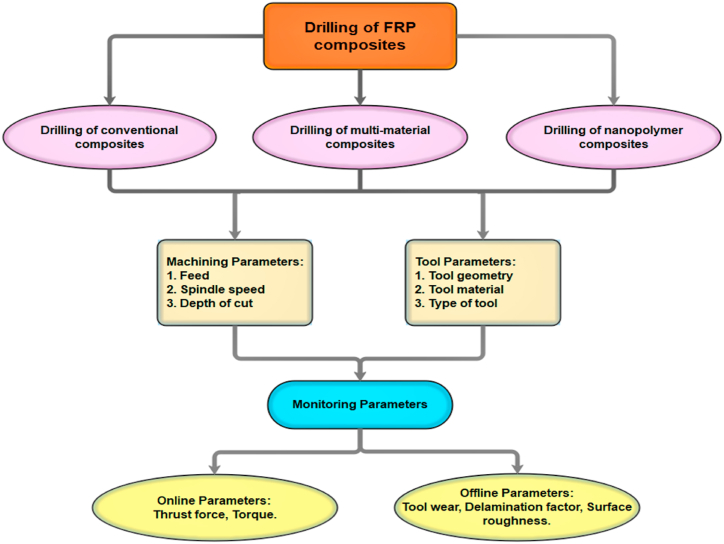
Schematic representation of FRP composites drilling parameters [24].

(1) *K*_*c*_ *= K*_*r*_*V*_*r*_ *+* *K*_*m*_*(1* − *V*_*r*_*)*

where *K*_*c*_ is thermal conductivity of FRP composite, *K*_*m*_ is the thermal conductivity of matrix material, *K*_*r*_ and *V*_*r*_ are the thermal conductivity and volume fraction of reinforcement fiber respectively. Along with the delamination, the sub-surface deformations also cause difficulty in FRP composites drilling through hole shrinkage, deformation of matrix, matrix crazing, and fiber pullouts. So, the improvement of both structural integrity and product performance of drilled holes is possible by reducing the delaminations and subsurface deformations through the optimum selection of drilling conditions, parameters, tool types, and geometries [20,21]. When a drill tool is used to drill a FRP laminate, it typically produces a thrust force along the longitudinal axis using the tool edge and then further deforms the surface of the workpiece due to friction. The deformation was uniform up to two plies of FRP composite laminate and further delamination was observed for extension of drill tool due to lower stiffness, deformation resistance, and smaller thickness of uncut chips. Hence the exit hole side of the laminate was found with more cracks propagation and matrix damage, which results in low surface integrity [22,23]. The schematic representation of FRP composites drilling parameters is illustrated in Fig. 1 [24],.

The tool’s abrasion phenomenon, where the flank and rake surfaces were subjected to rubbing action from hard surfaces, was the primary issue that surfaced during the drilling of FRP composites. Moreover, this abrasion phenomenon caused a reduction of machined surface performance for a long duration with reduced structural integrity. To avoid the defects raised in the FRP composites drilling, the conventional materials namely CFRP and GFRP were reinforced with nanomaterials like carbon nanotubes, polyamide-6, and carbon nanofibers for properties enhancement [25,26]. After drilling, the drill tools with diamond and carbon coatings function well in terms of tool life and wear. When drilling continued with a faster cutting speed and a slower feed rate, delamination in composite materials was typically seen to be at a minimum. Also, the lower delamination is possible with the tool having lower twist drill point angles [27,28]. The delamination factor (F_d_) was usually defined in two ways, one is the proportion of maximum diameter of the hole including a damaged area to the drill diameter and is illustrated in Eq. (2) [29,30]. In some literature, the delamination factor was defined as a ratio of delaminated/damaged area to the particular area of the hole, and it is illustrated in Eq. (3) [29,30].

(2) $$F_{d}=\frac{D_{\max}}{D_{d}}$$

(3) $$F_{d}=\frac{A_{\max}}{A_{0}}$$

where D_d_ and A_0_ are the diameter of the tool and area of the hole without any damages, whereas D_max_ and A_max_ are the maximum hole diameter and area including damages respectively. One of the key influencing factors in the drilling of FRP composites, where the quality of the drilled hole is crucial, is the tool geometry. When drilling polymer composites with tools made of tungsten carbide and high-speed steel, the two most crucial parameters are the feed rate and cutting speed. According to the literature review, the lowest frequent feed rate was 0.3 mm/rev, and the cutting speed ranged from 20 to 60 m/min. Due to the inclusion of strong filler metals in the polymer composites, the cutting speed is not constrained to the recommended range. Moreover, the maximum rotational speed is taken into account when using below 60 m/min of cutting speed [[31], [32], [33]]. The rise in cutting temperature caused by the increase in cutting speed over the ideal value caused the matrix material to soften. In order to prevent matrix deformation and increase the toughness and ductility of the resulting composites, the current trend involves the use of nanofillers as a viable candidate to change the direction of damage progression [34,35]. The interlaminar shear strength (ILSS) is a basic required property while drilling multilayered FRP composites. The structural components production like airframe, wing root fittings in the aerospace industry, and other stiffer elements are supposed to have maximum ILSS value. In this case, the higher value of ILSS prevents delamination during drilling and improves the structural integrity of the composites [36,37]. Several tactics are now mentioned in the literature for reducing delamination through the use of a smaller drill feed each rotation as well as using unique drill tool designs. The drilling of FRP composite with low drill feed per rotation doesn’t generate delaminations due to lower thrust forces below the critical value, although it is responsible for other types of damages like uncut fibers and burrs. In case of micro-drilling, the hole needs to be produced with micro dimensions and the feed per rotation should be low due to smaller tool dimensions [38,39]. The feed rate is a more critical thing that promotes damages and it is a required parameter to select the optimum feed rate to control the damages. Higher cutting speeds during drilling caused tool wear and increased torque and thrust forces. This causes larger damage rings and burrs at the exit and entrance. The combination of low drill point angle and higher speed are capable to avoid these problems. Some works are reported regarding the production of damage-free holes by selecting appropriate parameters using automation techniques instead of manual operations [40,41]. Its automated composite drilling technique is appropriate for large production and is operator-free. This automated drilling process includes torque sensors, temperature sensors, force sensors, processor control, and feed controls for the accurate production of holes in the FRP composites. In recent years, FRP composites are being used with unconventional machining processes like an ultrasonic, water jet, electric discharge, laser cutting, and electrochemical machining. Many studies related to CFRP and GFRP composites drilling quoted the difficulties in minimizing and controlling the surface roughness. After the machining of FRP composites, it was observed the fibers cut along and across their lay direction, also leaving partially disclosed fibers and deformed projecting fibers on the machined surface. The development of analytical models like empirical models, neural models, and classical models for determining the ideal circumstances for automated drilling also makes controlled drilling possible. Some possible problems might arise during conventional drilling of FRP composites like deflecting, skidding, and wandering of drill bits and it affects the hole position, also might cause dimensional inaccuracy. As the cutting edge enters the workpiece, this slows down the phenomena that causes the formation of steady forces. This continuous phenomenon throughout the plies causes tool damage at the last ply [[42], [43], [44]]. The impact of all these characteristics on the caliber of the drilled hole has, however, only received minimal research. The authors were inspired to review the characteristics and properties analysis of FRP composites because the majority of drilling studies on FRPCs in the earlier have concentrated on how to enhance the hole quality by maximizing processing conditions and there has been slight discussion on the correlation among drilling conditions, physical properties, and production techniques. The drilling feature of the workpieces manufactured from FRPCs is predicted to receive more emphasis from scientists and researchers as a specification in contrast to other qualities as a result of this research. Most of the reviews concentrates on recent developments in the respective field. But, this review clearly presents an overview of FRP composites drilling that had progressed from 2000 to 2021. The review covers the detailed analysis of different drilling conditions/parameters like thrust force, drill geometry, temperature, speed, and feed, also includes the post drilling analysis through delaminations, thermal damages, and surface roughness. Furthermore, the recent developments in carbon, glass, and natural fiber reinforced polymer composites are studied with both conventional and nonconventional drilling techniques. Several conclusions and future problems are inferred from this review based on the aforementioned findings.

## 2 Literature on FRP composites drilling (2000–2010)

Various types of drilling operations exist for FRP composites drilling, in that conventional drilling was more popular between the years 2000 and 2010. This section gives an overview regarding the drilling of FRP composites with multiple materials between the mentioned years. Some literature works are tabulated inTable 1. In GFRP composite drilling, the reinforcement of more glass fibers has an effective influence on chips formation than resin material. Delamination factor (DF) increased at the hole exit due to the increase in feed and flute number, while a decrease was seen at the hole entry. Both on the entry side and the exit side of the hole, the point angle and feed values immediately vary with DF. The bottom layers of composite experienced bigger shear stresses, hence the DF value was bigger at the hole exit than at the hole entrance. The 3 or 4 flute number, narrow point angle (<90°), and high cutting speed combination given a lower DF value at the hole entrance [52]. The experimental comparisons on conventional and vibration drilling revealed that the lower thrust was recorded in vibration drilling than the conventional process. When drilling CFRP and GFRP composites using both traditional and vibration drilling techniques, the thrust development was greater with the HSS drill than the carbide drill. The decrement trend of thrust force was observed with an increasing axial displacement of the drill tool, which was attributed to the generated accumulation of material cracks and fractures. This reduction of thrust force happened with vibration drilling and hence it is a suitable technique for producing microholes [53]. To get delamination-free holes and optimum drilling conditions, the semi-empirical relationship equations were reported in the literature. These equations were applied for aramid and carbon fiber reinforced epoxy composites to get optimum drilling parameters. This study quoted that the 6.35 mm drill diameter, 0.145 mm/rev of maximum feed were suitable for aramid/epoxy, carbon/epoxy composite to get good hole integrity, and this procedure is possible to follow with different materials with a variety of drill diameters [54]. Taguchi’s technique and various methods, such as analysis of variance (ANOVA), were used to find the best parameters for both K10 and HSS drill bits. The K10 helical flute drill tool was more efficient in producing fewer damages to the CFRP composite than K10 four flute carbide drill. The tool wear that occurred after drilling was not a significant one, but the wear was slightly more in the HSS drill about 0.012 mm along the flank surface. Finally, the confirmation tests obtained from different approaches revealed the delamination factor error within a range of 0.4–2% [55]. Enemuoh et al. [56] developed a statistical approach to get damage-free holes in carbon fiber reinforced thermoset composites. This approach is a relatively robust process and simple where the first optimization step involves the optimum parameter range selection with residing of global optimum values. This optimum parameter range was selected using ANOVA and analysis of means with the support of Taguchi’s orthogonal array. The process models were created based on this parameter range utilizing analytical models based on experimental data in the database and metal cutting theories. In the second step, the global optimum value is selected within the ranges provided in the first step and this step involves a multi-objective optimization criterion through the algorithm. This approach is suitable for the production of robust designs and helpful in getting a good surface finish and delamination-free hole in carbon/epoxy polymer composites.

**Table 1:** Overview of research works reported from 2000 to 2010.

FRP composites	Utilized drill	Drilling conditions	Inference	Study
Glass fiber/epoxy	Two fluted HSS drill	Speed- 500 to 2500 rpm, Feed rate (*f*)- 0.02, 0.04, 0.06 mm/rev.	The optimum thrust force obtained was 70 N and is in good agreement with analytical models. The maximum tool life obtained for the optimum conditions like 0.02 mm/rev (feed rate) and 18.85 m/min (cutting speed).	[45]
E-glass/epoxy	Steel tool, uncoated carbide tool, TiN coated carbide drill	Cutting speed (v_c_)- 55, 71, 86 m/min, *f-* 0.04-0.1-0.15, 0.2 mm/rev.	The maximum thrust force of 450 N was consumed to drill 1000 holes using steel drill, and low force was required for uncoated carbide drill. The uncoated carbide drill reached a thrust force of 150 N at the final drilling of 24,000 holes. The deviation in roundness was observed in steel drill holed composite, and for the uncoated carbide drill it was negligible.	[46]
Glass/epoxy	Brad and spur type	Spindle speed- 500, 1000, 1500 and 2000 rpm, feed rate- 150, 200, 250, 300 rpm.	The feed rate was the main influencing parameter in this study on roughness and delamination factor. The drill diameter had more influence on the delamination factor and less influence on the roughness value.	[47]
Carbon fiber/epoxy	Step drill	Feed rate- 0.01, 0.02, 0.03 mm/rev, Spindle speed- 800, 1000, 1200 rpm.	The optimum conditions such as 1200 rpm speed and 0.01 mm/rev of feed rate were adopted for drilling CFRP composites. Regression and the radial basis function network indicated with an errors of 0.3% and 28%, respectively. The parameters like stage ratio, feed rate and step angle were significantly affected the overall performance.	[48]
Jute/epoxy	Two facet twist drill, HSS drill, CoroDrill (854, 856)	Speed-750, 1250, 1750 rpm, Feed- 0.05, 0.1, 0.15 mm/rev.	The thrust forces were generated more with CoroDrill 854 drill than HSS and CoroDrill 856 drill. Whereas the delamination size was less with the HSS tool than other two. Jute fibers' coarse structure in the JFRP composite resulted in the smallest possible delamination size. This was attributed to less contact of fibers with the drill than the matrix.	[49]
Carbon/epoxy	K20 carbide drill	Cutting Speed-30, 38, 53, 80, 102 m/min, Feed rate-0.025, 0.05, 0.01 mm/rev.	The minimized delamination and thrust force was observed at 53 m/min of cutting speed and 0.025 mm/rev of feed rate. The thrust force was reduced by 12% for above feed rate, and 35% reduction was observed for the optimum cutting speed.	[50]
Glass fiber/epoxy	Cemented carbide drill (K 20)	Spindle speed- 4000, 8000, 40,000 rpm, feed speed- 1000, 3000, 6000, 9000 mm/min.	As per the tested conditions, the delamination was increased with increasing spindle speed, which might be due to increasing cutting temperature. The delamination was increased with feed at 4000 rpm and 8000 rpm speeds. Author suggested the higher spindle speed to get more material removal rate with minimum delamination.	[51]

**Fig. 2:**
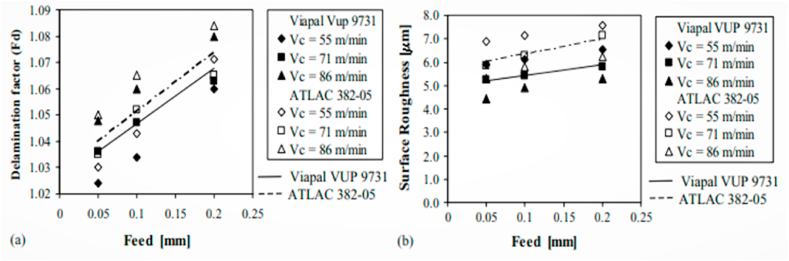
(a) Delamination factor [61], and (b) Surface roughness as a function of drilling parameters for different matrix reinforced composites [61] (Reproduced with the kind permission from Elsevier: Lic. No. 5492950447399).

The GFRP composite prepared with hand lay-up method was subjected drilling process by using two types of drills, namely “Stub length” K10 drill and “Brad and Spur” K10 drill. This experiment analysis reports the correlation between the feed rate, cutting velocity with thrust force, roughness, and cutting pressure. For “Stub length” and “Brad and Spur” K10 drills, the feed rate has a bigger impact on the thrust force by 93.7% and 94.5%, and on the cutting pressure by 93.2% and 99.1%, respectively. The damage analysis of the GFRP composite revealed that the “Brad and Spur” drill exhibit less damage than the “Stub Length” and it was measured in terms of damage factor. The measured roughness value was good at lower cutting velocity and more feed rate [57]. The Rule-based fuzzy logic model was an efficient one while predicting the thrust force values by using experimental data and could be applied for drilling parameters analysis in GFRP composites. The drill diameter and feed rate are the two factors that have the greatest influence on the generation of thrust force, while the impact of spindle speed was negligible and insignificant, according to an effective analysis of the various parameters on GFRP composites drilling using Pareto and traditional ANOVA [58]. The drilling defects and quality of the FRP composites were usually affected by cutting temperature. The continuous heat generation takes place while drilling of consecutive holes and it causes significant nominal bore diameter expansion. Concerning the temperature developed at the cutting edge, the damages that occurred in FRP composite were expressed through decomposing, pyrolysis, and melting. The increase in feed rate and cutting speed caused the cutting temperature to rise and had a substantial impact on the thermo-mechanical and physical properties [59]. The research work done by Palanikumar et al. [60] reports the influence of tool point angle during the drilling of glass/epoxy composites. Three alternative point angles (85°, 115°, and 130°) were used to study the drilled composite, and the 85° point angle produced the best findings. The increase of tool angle results in more delaminations and damages to the fiber composites. Moreover, the angles 115° and 130° show an almost similar tendency during the drilling process. Based on the findings, the author came to the conclusion that drilling GFRP composites at high speed while using a reduced point angle and feed resulted in a lower delamination factor. Davim et al. [61] studied the effect of different matrix materials (Viapal VUP 9731 and ATLAC 382-05) and processing parameters on the surface roughness, delamination factor (Fd) of drilled GFRP composites. The comparison of roughness and delamination factor for both varieties of composites as a function of drilling conditions is clearly shown in Fig. 2. The matrix ATLAC 382-05 reinforced composite shows a slightly more Fd value than the Viapal VUP 9731 matrix composite, which means the drill tool imposes more damage on ATLAC 382-05 composite than another one. With a faster cutting speed and a higher feed rate, the roughness value increased. Additionally, it was shown that Viapal VUP 9731 reinforced composite had a better surface quality than ATLAC 382-05 composite.

Mishra et al. [62] conducted a studies on unidirectional GFRP composite drilling process and calculated the residual strength drilled composite. The delaminations, cracks, debonding and melting phenomenon reduces the residual strength of GFRP composite. The accuracy of the model’s predictions is improved by optimizing drilling parameters such tool shape, feed, and speed using an artificial neural network. Murthy et al. [63] investigated the process parameters influence on the hole quality of drilled GFRP composites using design of experiments (DOE). The findings show that drill geometry, as opposed to drill size, has a significant impact on the quality of drilled holes. The surface roughness value was independent from fiber orientation and fiber volume during drilling process. The experimental findings revealed a minor impact of speed and feed on thrust and roughness. Drill angle (62% of the thrust force) was the primary factor, followed by material thickness, fiber volume, and drill size, all of which had equivalent effects. As compared to the sharp drills, the worn drills were allowed with low feed rate, and the damages can be avoided below this critical low feed rate. The higher thrust forces results in tool wear and this was responsible for delaminations followed by poor surface quality. The more thrust force was also results of faster axial speed [64].

## 3 Analysis of drilling conditions/parameters

### 3.1 Thrust force

**Fig. 3:**
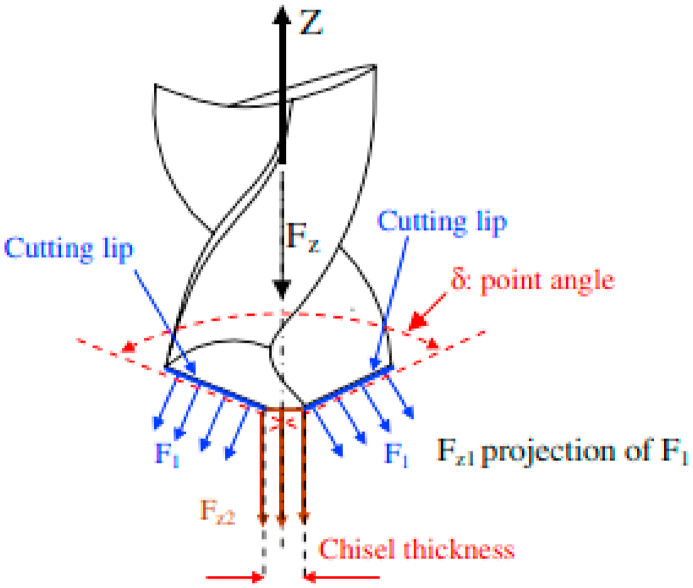
Decomposition of total thrust force (Reproduced with the kind permission from Elsevier: Lic. No. 5492950795141) [65].

The feed rate directly affects the thrust force, which was the main factor impacting the delaminations during drilling FRP composites. The overall resultant thrust force *(F*_*z*_*)* generated in the composite can be divided into two forces (*F*_*z1*_*, F*_*z2*_), where the *F*_*z1*_ force was attributed to generation of thrust force due to cutting lip contact with the composite and *F*_*z2*_ force owing to force generated due to chisel edge contact with the composite. The resultant force (*F*_*z*_ *= 2F*_*z1*_ + *F*_*z2*_) value was measured by drilling of FRP composite without the pilot hole. The force generated by the two cutting lips was denoted as *2F*_*z1*_ [65,66]. The clear decomposition of thrust forces is illustrated in Fig. 3 [65],. Thrust force was the lowest at the beginning of the drilling process, increased steadily, and reached its maximum value in the middle of the drilled hole. Further, the thrust force was reduced from the center hole drilling to the hole exit. Additionally, the drill bit exiting out of the opposite side of the composite caused the thrust force to decrease. Sisal/glass hybrid polymer composite drilling revealed this difference in thrust forces [67]. In every drilling process there is a possibility of determining “critical thrust force”, where there will be no damages and delaminations below this critical value. The thrust force reduction depends on the various parameters like tool material, tool geometry, FRP material, coolants and drilling parameters. In one of the study reported by Basmaci et al. [68] revealed the use of liquid nitrogen as a coolant effectively to ensure good machinability and production of damage free holes during drilling of CFRP composites. In some experiments, the thrust force was expressed as a quality character factor to notice the influence of drilling parameters especially for stretch and ratio of cutting velocity. Out of the five control parameters, the drill type, cutting velocity ratio, and feed rate have the greatest effects on thrust forces [69].

**Fig. 4:**
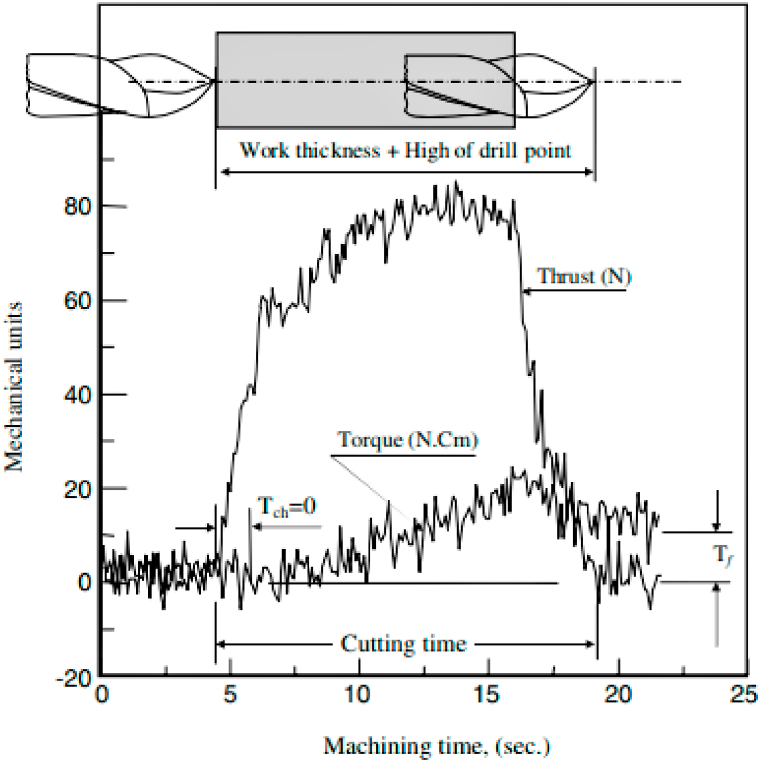
Thrust force and torque variations over a drilling cycle (Reproduced with the kind permission from Elsevier: Lic. No. 5492950997356) [70].

**Fig. 5:**
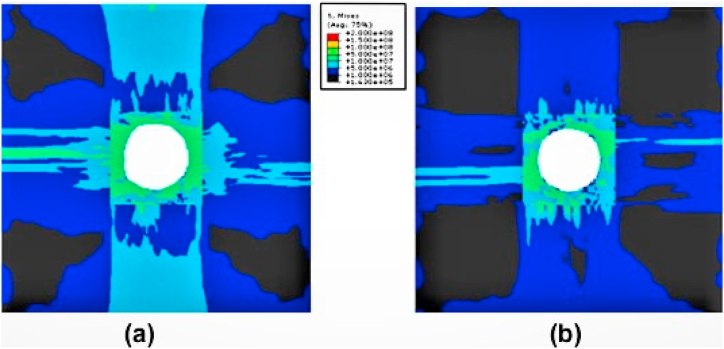
Stress distribution at CFRP workpiece using numerical prediction with (a) Twist drill [72], and (b) Step drill [72] (Reproduced with the kind permission from Elsevier: Lic. No. 5492951267831).

Khashaba et al. [70] reported the variation of thrust forces and torque concerned towards machining time during the drilling of GFRP composite. The variations are represented in Fig. 4. The initial thrust force generation is shown with immediate sharp peaks, followed by slow increment after the full engagement of the drilling tool. This sharp increment of thrust force has many reasons, such as the chisel edge having a negative rake angle results in more thrust force. This makes it difficult in drilling at the begging due to the negative rake angle and shows a sharp increment of thrust force. The common rake angle was positive at the outer drill diameter and negative near the drill center. This variation of rake angles in the same drill causes an increment of thrust force. Ramesh et al. [71] investigated the influence of different tool materials on the thrust force of sisal reinforced GFRP composite during the drilling process. The highest thrust force was produced for the HSS drill tool (264.32 N) at 0.08 mm/rev feed as opposed to solid carbide drill (218.56 N) tool and TiN coated solid carbide tool (238.58 N), and this experimental result was also associated with the variation phenomenon as shown in Fig. 5. Three different types of drills were used in the glass/epoxy composite drilling technique described in the literature to examine how thrust force affected delaminations. It was observed that there was no direct relation between the thrust force and delaminations, which means that the different types of drills were responsible for maximum thrust force and also it was responsible for secondary smaller delaminations. The carbide drill A1167A with three cutting edges and 150° point angle generated maximum thrust force while drilling glass/epoxy composite as compared to other carbide drills having two cutting edges and minimum point angles [73]. Mudhukrishnan et al. [74] studied the thrust force and delamination in glass/polypropylene composite using different types of drills. With an increase in feed rate and a drop in spindle speed, the drilling process’s imposed with thrust forces. The maximum value of thrust force recorded was 55–70 N for the HSS drill tool due to higher tool wear rate and lower hardness, whereas the solid carbide and tipped carbide drill generated lower thrust forces as compared to HSS drill. The drilling process using the HSS twist drill shows a rapid increment of thrust force up to 0.15 mm/rev of feed rate because of matrix clogging at the drill point.

### 3.2 Drill geometry

The drill bit’s point angle, cutting lip, chisel edge, and rake angle all had a big impact on how well the polymer composites did when drilling. The thrust forces were produced by the drill point angle, and their combined impact causes wear to advance. However, the thrust force is barely impacted by the angle of the drill tip on the new tool. The exact drill point angle is therefore one of the key factors when choosing a drill geometry, and occasionally it results in higher thrust force than is suitable for the drilling process [75,76]. The Brad and Spur drill with 118° point angle has numerous advantages like decrement in delamination and thrust force with limiting the chisel edge modification. The step drill geometry limits the usage of multiple bits for different sizes, and also helps in production of relatively good finished holes [75]. Whereas the twist drill geometry consumes low power, long life of cutting edge and lowers the operation time [74,75]. The helical-flute and four-flute drill geometry helps in effective removal of chips from the holes with the help of helical flutes, and these can be used for short chipping and hard materials [53]. The dagger drill and helicoidal drill geometries are suitable for drilling carbon fiber based materials with delamination free holes and industrial quality high speed drilling processes respectively [72,76].

Isbilir and Ghassemieh [72] conducted a numerical investigation on the influence of drill geometry on the drilling of CFRP composites. The developed numerical model was effectively captured the onset failure modes of composites. The step drill geometry performed better during the drilling operation than the twist drill in terms of both thrust force and torque values. Significant damages and delamination reduction was possible with using a step drill. Moreover, the stress distribution around the hole was more in the twist drill than the step drill. The execution of the step drill at the secondary cutting stage with the reduced contact area was to fault for this. The numerical prediction of stress distribution for different tool geometries is illustrated in Fig. 5 [72]. When drilling flax/PLA biocomposites interns with lower feed values, 2.6% push out and 3.6% peel up were observed, however the drill point effect at the exit and entry of the hole was decreased with a higher feed rate of approximately 0.76% and 1.93%, respectively. The created numerical model shows how drill point angle affects damage, and it was observed that increasing drill point angle caused damage to spread inside biocomposites [77]. The Reamer drill was the more satisfactory drill for the drilling of woven composites with higher cutting speed and lower feed rate. Reamer drills used for drilling CFRP composites have the smallest thrust forces and torque as well as the smallest delamination extensions (1.01–1.08). The special feature of this tool was the impact on thrust force was less for different values of cutting parameters. This tool worked well with tape-based materials when the drilling settings were adjusted to a quicker cutting speed and a lower feed rate. Yet the fraying flaw was the most prevalent and serious issue with this instrument. The drilling of woven CFRP composite with a Brad drill produces larger thrust forces and has inferior surface quality in other drill geometries [78]. The uncoated stepped drill geometry with a higher feed rate was capable to produce a maximum of nearly 2900 small diameter holes in CFRP composite with increased tool life, and also it gives lower thrust forces. The smaller chisel edge and the way the workpiece interacted with the step drill contributed to the decrease in thrust forces. Additionally, the feed rate increment decreased as the tool made more contact with the materials, which is why the reduction of abrasive action occurs at lower cutting temperatures [79]. Gemi and team [80] worked on effect of different drill types on drilling analysis of GFRP composites pipe applications. According to the study, twist drills occurred with larger thrust forces whereas brad center drills were generated with lower thrust forces. It might be preferable to utilize a support on the interior of the pipes while drilling process in order to minimize damages, keeping into mind the damage creation, particularly at the hole exit. Another interesting study [81] of coatings over drill tools has significantly affected the hole dimension and circularity. The coated drill also have a significant impact on drilling aeronautical distinct materials comprised of metal and composites because they may increase surface quality and extend tool life.

The unique work was published by Rakesh et al. [82] regarding the utilization of different solid and hollow shape tools (Twist drill, Jo drill, U shape, and Trepanning) for drilling of GFRP composites that were manufactured through hand lay-up technique. These hollow drills were particularly suitable for drilling laminated composite materials. When using a U-shape or Trepanning drill, the cutting action began at the tool’s outer edge, which put tension on the fibers throughout the drilling cycle and led to lower forces with induced damage. The U shape drill possesses 2.5 times lower thrust forces than the twist drill, hence authors not recommended a Twist drill for GFRP laminates drilling. The literature has reported on the impact of the cutting parameters when using a Brad drill bit to drill CFRP composite. In this criteria, the thrust force was gradually increased with the feed rate and decreased with the cutting speed. Contrarily, using Brad and Reamer drills, cutting speed had a minimal impact on thrust force during CFRP weave drilling while feed rate had no effect. The minimum thrust forces were observed with Reamer drills and maximum with Brad drills [83].

### 3.3 Temperature

Due to the lower values of the transition temperature and thermal conductivity of FRP composites, the temperature produced during the drilling operation was used. The heat generated around the drilling tool pulls down the stability of the matrix and causes thermal damages through rough and fuzzy cuts. On the other hand, matrix softening and decreased cutting forces were caused by the cutting temperature [84,85]. The ideal drilling area temperature for carbon fiber reinforced epoxy composites is greater than the resin’s brittle deformation temperature (T_b_) and lower than the fiber’s glass transition temperature (T_g_). When the drilling temperature was more than the T_0_, the anti-deformation ability and interfacial strength of carbon/epoxy composite was lower, which develops more delamination and surface roughness at the hole exit. Whereas in case of temperature lower than T_b_, the composite possesses a more brittle nature which enhanced the drilling force and led to exit delaminations. Hence the author suggested to maintain the drilling temperature range between the T_b_ and T_0_ to avoid the probable drilling damages [86]. Jessy et al. [87] developed a regression model for the analysis of drilling temperature in GFRP composites under different conditions like dry drilling, external coolant, and internal coolant. The linear equation for multiple regression has been developed using the experimental temperature and parameters response and Eq. (4) [87] is given below.

(4) T = c_o_ + c_1_ + (F) + c_2_(S) + c_3_(C.P) + … … … … + ε

where T-drilling temperature, F-feed, S-speed, C.P-Coolant pressure, ε-error, and c_o_,c_1_,c_2_,c_3_ are process parameters estimates. This equation was derived using the MINITAB 15 statistical software. The regression equations for drill temperature under dry, internal coolant and external coolant are represented in Eqs. (5) [87], (6) [87] and (7) [87] respectively.

**Fig. .6:**
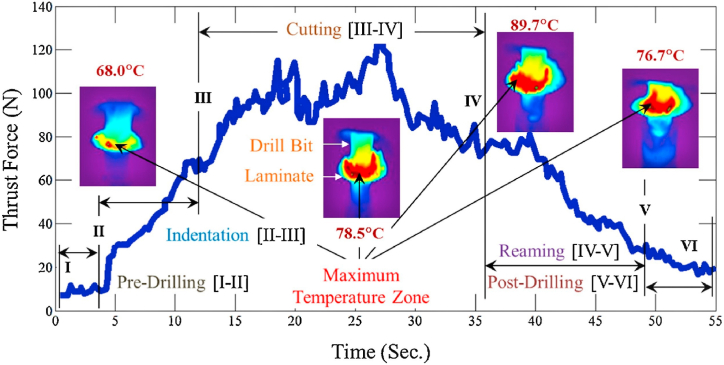
Correlation between thrust force and drilling temperature (Reproduced with the kind permission from Elsevier: Lic. No. 5492970858207) [90].

(5) T = 129 + 127(F) + 0.0209 (S)

(6) T = 22 + 437(F) + 0.0447(S) + 4.2(C)

(7) T = 37.7 + 22(F) + 0.0042(S) − 33.3(C)

where F-feed, S-speed, C-coolant. This model relates the cutting temperature as a function of feed and speed with the presence of coolants. This model aids in identifying the ideal circumstances for obtaining the lowest drill temperature to prevent thermal damage. The temperature variation plots of GFRP composite revealed that the temperature increment was mainly due to the increase of spindle speed. Another major factor that influences the cutting temperature was the type of drill bit. The HSS-R drill bit generates maximum temperature while drilling of GFRP composite, and TiN coated HSS drill lowers the temperature than HSS-R. The coating of TiN material on the drill bit shows a reduced drill temperature by 60% and ZrN coating results in 45% temperature reduction as compared to uncoated drill bits. The more heat transfer nature of the drill bit lowers the working temperature and reduced the damages that occur due to temperature generation at the interface [88]. The increase in the drilling temperature of carbon/epoxy composites results in the lower material stiffness induced by decrement of matrix modulus value. This lower material stiffness generates delaminations, cracks, and other damages to the composites. The authors suggested a temperature of 186 °C as a critical one to produce damage-free holes in carbon/epoxy composites. The transverse cracks were more at the hole exit due to drilling higher temperature. The temperature increment effect caused three modes of failures, such as the friction between matrix and fibers, and the reduction of thermal stability in matrix chemical and interfacial bonds. This was reported that the temperature increment might also changes the failure modes from matrix failure to interfacial bonds failure [89]. The drilling of lignocellulosic fiber-reinforced polymer composites investigation shows that laminate strengths are reduced when drilling temperatures are higher than the glass transition temperature. These variations in cutting temperature affect the tool life and hole quality. Fig. 6 [90], shows the relationship between drilling temperature and thrust force. The presented thermograms have different drilling stages, such as indentation stage (II–III), cutting stage (III–IV), reaming stage (IV–V), and finally the post drilling stage (V–VI). At the indentation stage, the cutting tip tries to extrude the top ply material, and hence thrust force signal is suddenly boosted in the graph. At this time, the ambient temperature was elevated from 21.9 to 68 °C. After the indentation stage, the cutting stage begins, and a further increase in temperature was noted from 68 to 78.5 °C. This cutting stage was important in the drilling process where the matrix and fiber materials were broken with maximum thrust forces and cutting temperature. Further, it was followed by the reaming stage where the thrust force signals were low. Finally, in the post drilling stage, the force and temperatures were stabilized and reached a minimum value [90].

Due to the reduced thermal conductivity of natural flax fibers, the quality of the drilled hole surface was lower in flax fiber reinforced epoxy composites. As a result, the heat that is produced builds up at the hole’s surface and softens the matrix material. The flax/epoxy composite drilling with twist drill shows a minimum cutting temperature, whereas CoroDrill 856 led to maximum temperature. The hole diametrical changes and more surface roughness was the results of cutting temperature increment [91]. When CFRP composite drilling was used, the temperature of the composite drastically decreased, increasing the material’s tensile strength and young’s modulus values. This makes the composite more fragile, stiffer, and brittle. Usually, the materials are more resistant to deformation at a lower temperature, hence the CFRP composite drilling under cryogenic conditions generates more thrust force as compared to drilling under dry conditions. Cryogenic coolant is used to produce high-quality holes and damage-free surfaces in CFRP composite by preventing a temperature increase at the cutting area. Overall, by lowering the heat in the cutting zone, drilling CFRP composite under cryogenic conditions enhances machinability [68]. The drilling of carbon/epoxy composite produces more cutting temperature and thrust forces than carbon/polyamide composites, which indicates that the thermoset-based composite is having poor machinability. In addition, regardless of the type of composite, the temperature signals typically change with drilling time [92].

### 3.4 Feed and speed

For FRP composites to produce damage-free holes, cutting parameters like feed and speed must be set at their ideal values. The type of material being used, the temperature at which it will work, and the type of drill being utilized will all influence how these parameters are chosen. The delamination factor was also dependent on feed and speed [93,94]. The delamination tendency of CFRP composite was more sensitive towards the feed rate changes, and non-linear variations were observed with cutting speed. Hence the delamination phenomenon was increased with increasing feed rate. The contour plot findings show that high cutting speed at decreased feed rate resulted in fewer delaminations, which highlights the significance of high-speed drilling in CFRP composites [95]. The surface integrity was the major thing that affects the drilling process of CFRP composites. Due to a reduced feed rate before the drill exit, a reduced surface roughness value of 4.04 μm was recorded at the bottom of the hole compared to all other sections. The type 2 CFRP composite laminate having 36 plies with 9.36 mm thickness shows a minimum surface roughness value of 1.16 μm at 90 m/min feed rate, and type 3 composite (40 plies, 10.4 mm thickness) gives a maximum roughness value of 2.47 μm [96]. Kevlar fiber reinforced polymer (KFRP) and abaca fiber reinforced polymer (AFRP) composite drilling behavior has been compared in a study that has been published in the literature. These composites were studied with 1000, 1500, 2000 rpm rotational speed and 30, 45, 60 m/min of feed. The thrust force was declined at 1000 rpm and 30 m/min of cutting conditions and this was correlated with the drilling of AFRP composites. Whereas the KFRP composite generates higher thrust force due to the requirement of higher speed and feeds as compared to AFRP composite [97]. The impact of speed, drill point angle and feed on the machining variables such as thrust force, delamination factor, and torque for bi-directional cotton reinforced polyester composite (BD-CPC) was reported by Chaudhary and Gohil [98]. During the BD-CPC composite, feed rate had a significant role in determining the thrust force. For BD-CPC composite drilling, it is crucial that the parameters lower feed, spindle speed, and point angle be set to 0.05 mm/rev, 4500 rpm, and 900, respectively, in order to reduce the thrust force. The value of torque was significantly affected by the interaction among feed and point angles. For drilling BD-CPC composite, the combination of 4500 rpm speed, 0.05 mm/rev of feed, and 90° point angle yields the lowest torque value. Merino-Pérez et al. [99] reported the influence of cutting speed and material constituents while conventional drilling of woven CFRP composite using WC-Co tools. In this experiment, the thermosetting matrix plays a major role while increasing the thrust force, and negligible impact was found on cutting speed. However, the torque generated during the drilling operation is mainly affected by the cutting speed. Roughness is primarily influenced by cutting speed than by feed rate.

## 4 Post drilling analysis

### 4.1 Delaminations

**Fig. 7:**
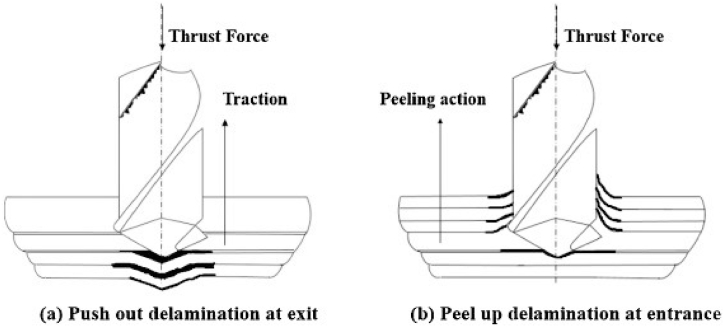
(a) Push-out delaminations at exit [100], and (b) Peel-up delaminations at entry [100] (Reproduced with the kind permission from Elsevier: Lic. No. 5492971044660).

Delaminations, peel-ups, and push-outs caused damage to FRP composites during both the entry and the exit of the drilled hole. The delamination process occurs through two mechanisms, such as push-outs and peel-ups respectively at the exit and the entrance of the hole (Fig. 7) [100,101]. The stacking sequence has more influence on the delaminations during and after the drilling process. The long CFRP composite shows more delamination at the interfaces of 0/±45° and 0/90°. Hence the fabricated laminate structure of these interfaces needs to be avoided at the free surfaces where there is a chance of large damaged zone development. The implementation of cohesive interactions during the modeling helps in reducing the delaminations in the FRP composites [102]. When drilling a CFRP composite, the use of compressed air cooling minimizes delamination at the hole entry, improving roughness and changing the resilience of the fibers in an air-cooled environment. This environment was also advantageous for fiber pull-outs reduction and damages during the requirement of higher cutting speeds. Whereas the delaminations propagation was more with higher push-down effects at the hole exit under dry cutting conditions. This may be connected to the altered material characteristics, particularly when polymer matrix is used at lower temperatures [103].

The complicated fatigue predictions reveal that the anisotropic and heterogeneous nature of FRP composites shows a complex stress state inside the constituents even with the light uniaxial loads. Hence it results in a matrix-fiber debonding and delaminations associated with other damages. This phenomenon was analyzed through kinetic theory of fracture to evaluate the intra-ply and inter-ply delaminations [104]. According to the traditional thermal rules, if faults in the composites caused discontinuities or gaps, the thermal properties of all composites altered. Similarly, the delaminations were identified as an air-filled defect that imposes resistance to heat flow inside the composite along the thickness direction. As a result, the delamination area grows linearly with an increase in the composite’s thermal resistance [105]. The delaminations were more susceptible to varied cutting parameters, and the combination of a lower feed rate and a greater spindle speed minimizes the delaminations, according to the jute reinforced polyester composite drilling experiment. Lower drill diameter and feed rates also made it possible to reduce the delamination factor [106]. The rice husk natural fiber reinforced polyester composite possesses lower delamination than the glass GFRP composite when subjected to the drilling process. Utilizing a twist drill with an 118° angle produces positive outcomes and reduces delamination. It was clear that the increased feed rate resulted in more delaminations when using the brad drill, where the spindle speed has a major impact on delaminations [107]. The delaminations were obvious in case of hybrid layered composites and it is evident with the literature reported by Gemi and coauthors [108] where the drilling study was conducted with hybrid glass and carbon layers reinforced composite for pipes application. The findings demonstrated that layering a carbon between two glass layers produced better outcomes in better mechanical characteristics, machinability, and lower damages. The stiffer carbon reinforcement serves as a supports and aids in preventing twisting deformity in the pipe.

### 4.2 Thermal damages

**Fig. 8:**
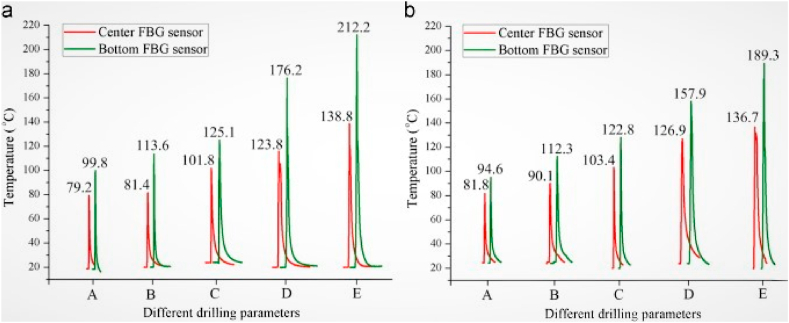
Drilling temperature of CFRP composites without (a) MWCNTs [113] and (b) with MWCNTs [113] (Reproduced with the kind permission from Elsevier: Lic. No. 5492971216203).

Due to the weaker transition and thermal conductivity of polymer composites, the heat produced during drilling was linked to thermal damages. This concentrated heat at the tool edge demolishes the stability of the matrix and causes thermal damages through rough and fuzzy cuts. Moreover, this cutting temperature increment was also responsible for lowering the cutting force and matrix softening [109,110]. The temperature measurement was recorded at the tooltip during the drilling and orbital drilling of Ti/CFRP/Al hybrid composite using a thermocouple. With a cutting speed of 40 m/min, orbital drilling produces a lower temperature of 82 °C and exhibits good surface quality without incurring heat damage. Hence it was very useful in installing the thermocouple at the tooltip for direct measurement of temperature to avoid thermal damages [111]. The FEM simulation of CFRP orthogonal cutting was accounted for thermal damages throughout the different layers up to the temperature of 150 °C. Due to the anisotropic thermal conductivity shown by the matrix and fibers, the varied fiber orientations have a significant impact on the temperature distribution. The friction generated at the interface causes more thermal damages, especially for the 0° and 45° orientations, and this his could be avoided by applying a proper coating to the drill bit [112]. The loading of carbon nanotubes in carbon/epoxy composite and microwave curing avoids the thermal damages induced during the drilling process. The neat comparison of drilling temperatures between without and with MWCNTs is illustrated in Fig. 8. The thermal damages that occurred in the filled composite were low as compared to unfilled composite, which was attributed to the absorption of drilling temperature by MWCNTs instead of hybrid composites. In comparison to conventional composite materials, these composites record drilling temperatures that are 23 °C lower than their glass transition temperature. Hence the MWCNTs modification results in lower thermal damages and higher drilling performance [113]. The frictional force between the drilling tool and the drilled hole wall surface causes the thermal damages to increase. Due to the lower beginning temperature when the tool is introduced to the workpiece, the assessment of thermal damages is considerably more important at the exit plane than the entry plane. With the passage of tool inside the composite, the temperature was gradually increased till the hole exit area with more thermal damages [114].

### 4.3 Surface roughness

When drilling bidirectional CFRP composites, the drill diameter had the greatest impact on the surface roughness, followed by other factors like spindle speed and feed rate. This is one of the surface quality measuring parameters and has more importance in structural components. During the process planning, this is one of the serious parameter while choosing the machine elements and suitable parameters selection. Numerous studies found that surface roughness and thrust force varied inversely with cutting velocity and directly with feed rate [115,116]. The maximum cutting speed and low feed rate led to less aggressive fracture, and it was easy to handle due to low strain rate. Hence it gives fine surface roughness and is recommended to set at low feed rate. The surface roughness results obtained from drilled GFRP composite revealed that the hole produced with carbide tipped and HSS drill has more surface roughness than the solid carbide (eight facets) drill. This was owing to the poor surface finish obtained from those two drills that might consecutively encounter fiber and resin in the GFRP materials [117]. When employing a solid carbide drill with the ideal settings of 0.05 mm/rev feed rate and 2500 rpm spindle speed, the drilled holes in glass/polypropylene composite produce better surface quality and dimensional fits. These good results are owing to carbide drills that are strong and hard enough to drill the FRP materials effectively [118]. According to the statistical and experimental examination of the hybrid carbon/glass composite, the feed was the main determinant of surface roughness, followed by speed and drill geometry. The authors suggested optimum parameters like 7500 rpm speed, lower point angle, and 0.08 mm/rev of feed to get the maximum quality of holes without altering production rate and tool life. The regression model used in this experiment was adequate for the prediction of response obtained from the surface roughness for hybrid composites [119]. Konneh et al. [120] experimented on surface roughness analysis of drilled CFRP composites by utilizing the diamond coated drill tool. With rotational speed and feed rate values of 5062 rpm and 180 mm/min, respectively, the lower value of surface roughness was reached about 0.90 m. Whereas the maximum value of surface roughness around 4.630 μm was recorded for the rotational speed and feed rate value of 537 rpm and 180 mm/min respectively. It means that a higher rotational speed was required to get a low finish during CFRP composites drilling. Interesting work was reported by Álvarez-Alcón et al. [121] reported the impact of cutting parameters on the surface roughness through multiple sensors monitoring. The best quality of holes with minimum roughness was obtained for the cutting velocity of 105 m/min and a feed rate of 300 mm/min. Other than these optimum parameters, the hole roughness values were enhanced by 21–140%.

## 5 Recent developments in FRP composites drilling (2011–2021)

### 5.1 Carbon fiber reinforced polymer (CFRP) composites

The CFRP composites have gained more importance in automotive, aerospace, construction, and other industrial sectors due to their superior characteristics. Sometimes these are subjected to the manufacturing of structural components where the machining processes are required to meet the design standards. The CFRP composites possess abrasive nature, inhomogeneity, and anisotropic nature, hence the machining process is associated with poor surface quality, delaminations, tool wear, and dimensional inaccuracies [122,123]. Shunmugesh et al. [124] studied the machining parameters optimization for drilling CFRP composites and analyzed the post drilling parameters like delamination, surface roughness, and dimensional accuracy. The findings show that both feed rate and cutting speed contributed equally to the reduction of delamination factor and roughness. The circularity of the hole was decreased with increasing feed rate to 0.1 mm/rev from 0.025 mm/rev. The optimum parameters to minimize the circularity and delaminations were 0.025 mm/rev feed rate, 50 m/min cutting speed, and solid carbide (TiAlN coated) drill bit. Due to material fissures and partially damaged fibers, the CFRP composite’s surface roughness was increased. The defect study conducted by Qiu et al. [125] for CFRP drilled composite shows that the delamination phenomenon was majorly influenced by spindle speed and then followed by feed rate. The optimum selection of lower feed rate and high-speed results in minimized delaminations and good surface quality. When using a brad point drill, the best speed and feed rates are 10,000 revs per minute and 0.01 mm per revolution, respectively, which results in the least amount of burrs and good drilling performance. When using a multifacet drill, the feed rate had a significant impact on the thrust force, and the best hole quality was attained at a feed rate of 0.004 mm/rev and a speed of 4000 rpm. Nagaraj et al. [126] worked on the drilling performance analysis of CFRP composite under cryogenic, MQL, and dry atmospheres. In comparison to the force produced in a dry environment, the thrust force produced in a cryogenic environment was 36% greater. This was attributable to the composite’s increased tensile strength and young’s modulus in a cryogenic atmosphere. Dry drilling causes matrix softening with little thrust force generation since it operates at higher temperatures. Under the MQL environment, the thrust force generated was 17.6% more than the dry drilling because the upward flow of lubricant causes more pressure for chips flow, and hence results in increased thrust force. This was correlated to the delaminations, that is cryogenic drilling induces 16% more delaminations than dry drilling due to maximum thrust force. The AlCrN coated tool done a favorable thing for the drilling of CFRP composite at higher speeds. This generates continuous and long chips with a lower possibility of material cogging thereby giving good machining performance than in HSS drilling. The initial flank wear that observed in the coated tool was negligible until the 15th hole drilling with a speed of 1300 rev/min, further it was observed significantly when the tool wear begins to occur till the final experimentation, and follows the similar trend of the HSS tool. The occurrence of more uncut fibers was found to be the cause of the corner edge wear observed in the drill more frequently in HSS than in the AlCrN coated tool [127]. During the low feed cutting, the cutting resistance was more due to uncut fibers thickness and it results in more shear forces which led to vibrations. Hence it was difficult to get exact hole diameter at lower feeds and better hole diameter accuracy could be obtained at a higher feed rate [128]. Due to maximum thrust forces reported at high and low feed rate values, which were directly related to laminations in the laminated structures, traditional drilling of CFRP composite utilizing a twist drill was not acceptable to drill laminated structures. The least delamination damages were observed with step drills along with good surface finish in this experimental work. Hence the step drill was recommended for CFRP laminates drilling [129]. When the ratio of chip thickness to cutting edge radius is less than unity when microdrilling CFRP composite, the size effect seems to be more pronounced. The installation of the drilling process should be careful to balance the radial forces generated on the two sides of the cutting edge and the resultant force should be zero. But it was difficult to get balanced forces on both sides and hence it develops inhomogeneity in the drilled holes. The longitudinal direction of the cutting force applied to the composite was greater than the transverse direction. This was due to the consequent layers of carbon and epoxy material having anisotropic properties [130].

**Fig. 9:**
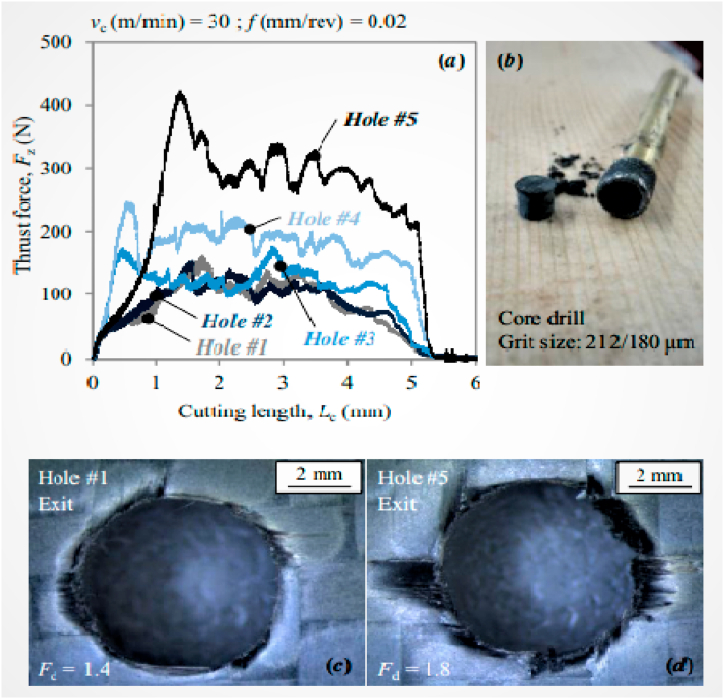
(a) Thrust force elevation graph [136], (b) Tool obstruction [136], (c, d) Influence on push-down delaminations [136] (Reproduced with the kind permission from Elsevier: Lic. No. 5492980157561).

Catche et al. [131] analyzed the hole wall defects raised in the CFRP composites drilling process. The primary drilling defect observed was sharp micro-cavities along with the ply thickness, and were observed at −45° orientation relative to tool rotation and ply direction. The uncut fibers were protruded and simply crushed during the drilling process, while the cavities formed were the beginning stage of delaminations and cracking. The CFRP drilling under LN2 cryogenic coolant shows an improved machinability and also reduces the tool wear by 30% with improved tool life. In order to prevent the development of a built-up edge, this cryogenic drilling reduced friction, raised the temperature, and provided excellent lubrication. In comparison to the surface that had been dry machined, the surface that had been obtained for the borehole was brighter, and the surface’s quality had increased by over 31%. The produced delamination damages under cryogenic machining were not fluctuating, where this fluctuation was commonly observed in dry drilling. The thrust forces generated were more in cryogenic machining, which was nearly 61% more than the dry drilling [132]. The induced delaminations during the drilling process were reduced the tensile strength, and further reduction was observed with increasing delamination factor. The strength was reduced by 15% for a delamination factor value of 3, whereas 5–10% of strength reduction was observed at 1.8 delamination factor. This reduction was due to the primary failure of outer plies where these were incapable to transfer stress between the following plies [133]. The numerical model developed for the analysis of woven CFRP composite drilling shows that the highest temperature was recorded at the drilled hole wall, especially at near to the hole exit where the delaminations were commonly observed. When there is excessive wear, the generated wall temperature was higher with the used tool than it was with the new tool, pushing the probability of faults at the hole exit. The maximum temperature recorded below the machined surface was around 104 °C and this was the critical point for the drilling process. Sometimes the worn tools are capable to produce a wall temperature of more than 180 °C [134]. There are many benefits to using a helical groove drill for clean drilling CFRP composite, including the removal of burrs at the entrance and exit of the hole during both upward and downward feeds by the bottom cutting edge. Hence even after the 100 holes drilling actions, the burrs were not found in the holes. The helical groove edge was responsible for getting more surface integrity at the holes inner surface. The uncut fibers and attached fibers inside the surface were also removed due to this action. With a helical groove drill, the ideal feed rate (0.0025 mm/rev) and spindle speed (800 rpm) resulted in a lower value for surface roughness [135]. When drilling CFRP composite, a core drill covered with coarse diamond particles produced better results than one coated with fine diamond particles in terms of torque, delaminations, and thrust force. In the multiple holes drilling phenomenon, the internal geometry of the tool was altered by workpiece material and it is illustrated in Fig. 9. As a result, the hole’s exit delaminations increase in number and the thrust force [136]. Heberger et al. [137] analyzed the effect of different machining strategies like circular milling, conventional drilling, and industrial point of drilling on the strength and quality of the rivet holes. The tensile results revealed that the rivet connection strength was directly related to the drilled hole quality. The delamination factor during drilling was discovered to be high due to drill tool movement along the spindle direction, which results in increased feed forces, and the helix tool angle, which promotes fiber peel-up. Moreover, the unfavorable angle between fibers and tool causes uncut and protrusion of fibers. Among the three strategies, the conventional drilling has maximum fiber protrusion and delamination factor. The experiment on drilling of quasi-isotropic CFRP composite under dry environment revealed that the maximum torque and thrust forces were increased with the feed values because of feed dependency on thickness of the undeformed chip. Because of the thermal expansion and the instabilities of the tool and workpiece, the hole diameter was larger than intended at higher speeds and lower feeds. The surface profilometer device was utilized to measure surface roughness value, and this was highly dependent on fiber pull-outs length than the pull-outs depth [138]. Recently, many cryogenic machining studies on CFRP composites has gained much interest due to feasible quality over machined surfaces. Morkavuk et al. [139] utilized liquid nitrogen as a cryogenic coolant for CFRP composites machining and studied the mechanical properties. Chip breakability was enhanced by cryogenic freezing, which rendered the specimen’s structure brittle and stopped heat degradation to the machined CFRP surface. Cryogenic machining exhibited lower levels of damage development than dry machining, as observed in SEM imaging [140].

### 5.2 Glass fiber reinforced polymer (GFRP) composites

**Fig. 10:**
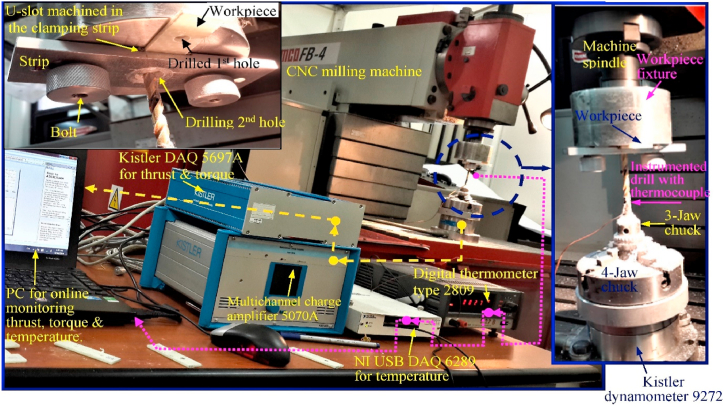
Experimental setup for the measurement of temperature, torque and thrust force (Reproduced with the kind permission from Elsevier: Lic. No. 5492980468378) [148].

The GFRP composite materials possess higher specific stiffness, specific strength, lightweight materials, good corrosion resistance, and other superior properties that are suitable for potential engineering and construction applications. One of the most popular areas of research right now is the machining of these composite materials, and more studies have been published in the literature [141,142]. The delaminations observed in GFRP composite drilling were more at 500 mm/min of feed rate instead of 100 mm/min because of more thrust force. The lower feed rate values led to better hole quality and it was observed through micrographs. The push-out movement was seen on the hole exit side as a result of delaminations, which caused the peripheral to emerge from the fibers. The uncut of fibers and frayed action led to protrusion of fibers. During the drilling process, the axial direction force exerted through the drill slope creates a delamination zone by splitting out the laminas inside the composite [143]. The Rotary mode ultrasonic drilling (RMUD) shows a superior drilling performance with improved MRR than the conventional UD during the drilling of glass/epoxy composite using both hollow and solid tools. More improvement in MRR was observed with the hollow tool than the solid tool. This MRR value was linearly increased with increasing slurry concentration, abrasive grits size, and power rating. All of these factors do, however, greatly reduce the surface roughness of the drilled holes. The tool wear rate during GFRP drilling was directly correlated with the MRR [144]. The correlation between feed, spindle speed, and thrust force throughout the experimental analysis of GFRP composite drilling was reported by Kumar and Sing [145]. It was shown that increasing the feed rate causes increased push forces, whereas increasing the spindle speed causes a reduction in thrust forces. It was attributed to more impact of drills on the GFRP composites due to the higher feed rate. The solid carbide tool having four cutting edges helps in easy removal of materials with lower thrust force at 0.1 mm/rev and 2000 rpm speed. The higher spindle speed led to more surface roughness due to the built-up edge formations. The addition of graphite powder filler into glass/vinyl ester composite increases the thrust forces during the drilling process as compared to unfilled composite. These filler particles cause more abrasion, tool wear and change the machining characteristics. The drilled hole in the unfilled composite has a better surface finish for the optimum parameters of 355 rpm speed and 0.12 mm/rev feed rate. Hence the addition of graphite fillers has no significant positive effect on the drilling process [146]. The drill tool acts as a punching element that penetrates the composite laminating instead of cutting it while drilling a GFRP composite with a greater feed (0.45 mm/rev). The matrix-fiber interfaces were destructed by more drill pre-wear due to increasing thrust forces. The abrasive action between tool and fibers caused partial shearing of fibers and hence results in poor surface finish [147]. The drilling of thin epoxy/glass composite with 16.3 m/min of low-speed results in higher thrust force, torque, delaminations, and lower temperature as compared to drilling speed of 65.3 m/min. The drilling of these composites nearer to glass transition temperature was helped in the reduction of delaminations, thrust forces and gives better bearing strength to the composite. Fig. 10 [148], depicts the competition for positioning to measure torque, temperature, and thrust force during the drilling operation. The action of uncut layer push down by the chisel edge results in the propagation of cracks inside the materials. The continuous progress of the drilling process caused an increase in thickness of the peeled-up layer and hence shows bending resistance [148]. Jena and Kumar [149] analyzed the drilling characteristics of clamshell filler reinforced GFRP composite. The combination of 1000 rpm speed, 0.04 mm/rev feed, and 6 mm drill diameter were optimum to get a lower delamination factor (F_d-in_) of GFRP composite. Whereas the lowered delamination factor (F_d-out_) was achieved for the optimum parameters like feed, speed, and drill diameter of 0.12 mm/rev, 1200 rpm, and 6 mm respectively. The addition of 20 wt% filler lowers the surface roughness, and this roughness was highly dependent on the tool diameter.

Palanikumar et al. [150] reported an impact of drill diameter on the feed rate during glass/polypropylene composite drilling process. It was observed that the increment of drill tool diameter slightly causes an increment of force during the drilling process. The variation of tool diameter led to increment in contact area with more heat generation, further it results in more thrust. The spindle speed increment led to matrix softening thereby minimizing the thrust force. The topographical surface analysis shows the presence of voids inside the matrix due to drilling action. Moreover, the bottom layers of the composite were observed with some variations due to compressive action and thrust during the drilling process. The drilling of glass/polycarbonate composite revealed that the damages and thrust forces were enhanced for all the three different diameters, such as 6, 9, and 12 mm. The hole size was also an important parameter for the development of thrust forces, which means that the higher hole size causes more thrust force. Along with this, the composite laminate thickness was directly varied with the thrust force [151]. The nanoclay addition to glass/polyester composite increased the torque during the drilling process and it was less for lower reinforcement (1, 2 wt%). Additionally, it was reported that the torque increase was seen after the feed rate was increased from 0.045 mm/rev to 0.1 mm/rev. Delaminations occurred in this experiment before the eventual failure of the composite, and the delamination factor value recorded for this composite was closer to unity, suggesting that the drilling procedure had less of an impact on the surface roughness of the composite [152]. Ficici et al. [153] analyzed the effect of tool material and drilling parameters on the glass fiber reinforced polyphthalamide (PPA) composites. The findings of the drilling test show that as the feed rate was raised and the cutting speed was elevated, the delamination factor reduced. Lower surface roughness values are produced by carbide tool drill bits, and lower feed rates have a greater impact on surface roughness. The investigation of the drilled surface revealed the existence of sheared fibers, fissures, and fiber pull-outs.

### 5.3 Natural fiber reinforced polymer (NFRP) composites

Due to their renewability, recyclability, low density, cost-effectiveness, and environmental friendliness, natural fiber-reinforced polymer (NFRP) composites are now widely used in a variety of industrial applications. The drilling of NFRP composite is a crucial one for advanced industrial applications due to critical dimensional tolerance requirements. The disadvantages associated with natural fibers such as low thermal conductivity, fibers shrinkage, incompatibility with polymer matrices, and low working temperature makes a drilling process as critical operation [154,155]. Different drill geometries have an impact on the sisal-reinforced polypropylene composite’s drilling performance (trepanning and twist). The drilling force was majorly affected by drill point geometry and cutting speed has less impact during the drilling of sisal/PP composites. Lower thrust forces than those produced by a twist drill were used to drill these NFRPs with a trepanning instrument. The hollow drill cutting mechanism was compatible with the drilling of sisal/PP composites. The continuous decrement of torque value was observed with the trepanning tool, and it was nearer to a constant value with cutting speed increment [156]. The drilled sisal/epoxy composite was produced with discontinuous chips, whereas the sisal/PP composite was produced with continuous chips. The production of chips is not significantly influenced by the drill geometry, but ring-shaped chips have been seen when feed and speed are reduced. For both sisal/epoxy and sisal/PP composites, the drilling force magnitude reduced with increasing spindle speed and surged with increasing feed rate. Sisal/PP composite drilling produced less torque than sisal/epoxy composite drilling. When compared to step drill and four facet drill geometry, the parabolic drill used with these composites exhibits low torque and thrust force. Debonding, fiber pull-outs, and smearing were found in this experiment, however there were no delaminations at the entry or exit [157]. Chegdani and Mansori [158] reported the influence of different drilling tool coatings on the flax fiber reinforced PP composite. The experimental results reveals that the diamond coating shows a progressively worse behavior on machinability than the titanium diboride (TiB_2_) coated tool. The thickness of the diamond coating increases intrinsic friction response and radius of tool cutting edge. Even with the maximum intrinsic friction response, the diamond-coated tool was exhibited by the lowest drilling friction. The uncoated drill tool exhibits maximum friction for all the cutting speed and feed as compared to uncoated tools. This tribological multiscale behavior was significantly affected by the nature of coated material at the interface of material surface and tool edge. The drilled hole quality observed on the flax reinforced biocomposites was highly dependent on the input parameters such as tool diameter, feed rate, and spindle speed. The behavior of natural flax fiber was different when subjected to thrust force due to its soft nature and higher cellulose percentage, thus more amount of energy dissipated through the deformation of the fibers when the fibers were in contact with a rigid drill tool. The high cutting speed drilling generates enormous amount of heat and natural fibers were unable to transfer this generated heat fully because of low thermal conductivity and transition temperature [159].

The tensile strength achieved in drilled flax fiber-reinforced composite was between 30 and 35 MPa, which was half times lesser than original composites without any holes. This shows that the composite strength was more with undrilled composite than the drilled one. This was due to the stress concentration around the drilled hole, thus reducing the overall strength when subjected to higher loads. The delamination factors recorded for this composite at the top and bottom surfaces were 1.29 and <1.20 respectively. This indicates that the delamination damages were more at the top surface than the bottom surface [160]. Vinayagamoorthy et al. [161] reported the optimum drilling conditions for Vetiveria zizanioides fiber reinforced polymer composites. The 2800 rpm speed, 0.2 mm/rev feed, and optimum point angles (80° and 120°) were optimum to get a minimum roughness value of 3.06 μm. A minor increase was seen with the elevation of point angle and feed, and the wall roughness within the hole was steadily increased with speed. The interaction of the cutting variables affects the wall’s roughness significantly as well. In comparison to non-woven coir/polyester composite, treated coir woven fiber reinforced polyester composite has a decreased delamination factor value. It was also reported that the delamination factor noted in the coir/polyester composite was low as compared to the GFRP composite [162]. The optimal drilling process parameters, such as drill diameter, feed rate, and spindle speed of 6 mm, 50 mm/min, and 3000 rpm, are revealed by the investigation of the signal-to-noise ratio for unidirectional jute reinforced composite. The drill diameter, feed rate, and spindle speed parameters each contributed 88.19%, 7.64%, and 2.62% of the variations and delaminations values, respectively, according to the results of the ANOVA results analysis [163]. The point angle and feed rate parameters during the drilling process were shown to have a substantial impact on the thrust force according to the drilling tests on sisal fiber-reinforced composite with coconut shell powder. The predrilled hole had to have a diameter of at least 0.4 mm in order to noticeably reduce thrust force. Additionally, the thrust force rises as a result of the larger volume fraction of sisal fibers [164].

## 6 Drilling of FRP composites using nonconventional drilling techniques

For FRP composites drilling, non-traditional drilling techniques are now employed to improve machining quality and prevent delaminations, fiber peel-up, and push-outs. The methods such as abrasive water jet drilling (AWJD), laser drilling, electrochemical drilling, electro discharge drilling, and ultrasonic drilling were employed for FRP composites drilling. The requirement of higher dimensional accuracy, speed, and more efficiency motivates the manufacturing industries to go with nonconventional techniques [165,166]. Geier et al. [167] performed a drilling studies on unidirectional CFRP composite using a three-axis CNC machine (Kondia B640). When compared to other feed rates, it has been found that the drilled hole with the highest feed rate of 300 mm/min displays a very low level of uncut fibers. Furthermore, the uncut fibers were reduced with feed rate having cutting speeds within the range of 50–100 m/min. The 105 m/min cutting velocity and 70 mm/min feed rate were found to be the best settings for minimizing thrust forces, while 88 m/min cutting speed and 300 mm/min feed rate reduced the growth of uncut fibers. The burrs generated during CFRP composites drilling were removed by electric discharge machining (EDM), and steel, aluminum, brass, and coppers were used as deburring electrodes. In these electrodes, the copper electrode shows a good performance towards MRR and deburring time. There was no morphological changes observed in the hole wall surface after the EDM process, and another advantage was the range of machining was easily controlled by adjusting the spark gap [168]. When drilling GFRP composite utilizing AWJD, the standoff distance and angle of fiber orientation had the biggest effects on dimensional instability and surface roughness. Beyond the critical limit, the surface roughness reduced as a result of the smooth cutting operation of the particles with more kinetic energy. Surface roughness was increased by the abrasive flow and hydraulic pressure increment up to this point. The reduced angle of fiber orientation and standoff distance with the increased abrasive flow and jet pressure led to better dimensional accuracy and hole surface finish [169]. The outward peeling of fibers in the CFRP drilled composite with AWJD revealed the delaminations at the hole entrance and exit. For the different angles of fiber orientations, the delaminations were more at the entry than at the exit side. Water flowability and stand-off distance had the greatest effects on surface roughness, with maximum effects of 87–88.1% and 7.2–11.4%, respectively. The drilled surface observations show that the abrasive particles cling to the surface of the hole’s gaps and fissures. The reduced surface roughness of around 0.99 μm was obtained for +45°/-45° fiber-oriented composite at a water pressure of 5300 bar, stand-off distance of 1 mm, and feed rate of 3000 mm/min for 12 mm hole diameter [15]. When the hole was drilled utilizing laser technology, the glass/epoxy composite’s bearing strength was decreased. The heat-affected zone (HAZ) that is formed around the hole has an impact on the holed composite’s bearing strength. The increased area of HAZ caused the decrement of bearing strength. In this laser drilling process, the fiber breaking was the major defect raises in the drilled composite [170]. The laser drilling employed on GFRP and aramid fiber-reinforced polymer (AFRP) composite shows dark heat-damaged regions inside the drilled hole surface. Because of resin pyrolysis at the surface and the various decomposition temperature of resin and fiber, this black damage was seen. More amount of fiber volume removal was done with shallow drilling depth using laser technology, which was due to the higher laser light density near the drilling point on the composite surface. For the specific depth of the hole, the drilling requirement of irradiation frequency was two times more for aramid layer reinforcement and four to five times required for glass layer reinforced composite. The maximum efficiency of drilling was obtained with AFRP composite than GFRP, which was due to the presence of organic materials in the AFRP and mineral materials in GFRP composites. The variations of decomposition temperature between fiber and resin also cause roughness inside the hole surface [171]. The ultrafast laser drilling was an effective technique for drilling CFRP composite to minimize the HAZ area. In this technique, the femtosecond laser was utilized instead of the picosecond laser because of the smaller pulse width and it helped in getting a good surface hole quality in the composite. The dimensions at the hole’s entry and exit points increased as a result of increases in parameters like feed depth and laser power. By considering all these developments, the laser drilling with femtosecond laser delivered a highly précised holes, thereby promising higher joint strength and less weakened structures [172]. The increase of kenaf/HDPE composite thickness to 6.7 mm from 3 mm led to the reduction of HAZ width by 5.79% and kerf taper angles by 9.33% during the CO_2_ laser drilling process. The laser beam power of 135 W with a cutting speed of 2 mm/s results in the maximum width of HAZ (0.8 mm). Further variations of cutting speed and laser power by 4 mm/s and 120 W respectively led to an increase of HAZ width by 53.84% [173].

## 7 Challenges and future scope

The components that make up FRP composites, notably the reinforcing properties that either make machining easier or more difficult, determine how machinable they are during drilling process. When these FRP composites were subjected to the drilling process, it exerts a higher thrust force and causes damages to the composites like delaminations, internal cracks, debonding, and sometimes led to material failure [174,175]. The higher thrust force generation led to more temperature at the tool-workpiece interface and cause composites deformation with dimensional inaccuracies in the drilled holes. During drilling process, the amount of torque and thrust forces generated were directly correlated to the proportion of natural fibers reinforcement in the composites. This was caused by a rise in composite static strength brought on by the presence of natural fibers, which reduces resistance to drilling. When drilling tough FRP composites, where the temperature and thrust forces are higher, the tool softens and sticks to the material as a result of abrasion wear [[176], [177], [178]]. In some cases, the chemical wear was observed due to chemical reactions among cutting fluid, workpiece, and the tool. The heat generated during drilling causes strength alleviates and oxidation at the cutting tooltip. Due to the various characteristics of the matrix and reinforcement materials, it is important to assess the parameter selection before drilling of distinct FRP composites. The major common problem that was raised during drilling was chips clogging with the tool material during either rotation of workpiece or tool. Hence this needs to be avoided by selecting suitable drill tool geometries and selecting a particular material for specific tools [179,180]. According to several studies, there may be a significant effort made in the future to integrate analytical and simulation-oriented methodologies. The sophisticated in-process observation techniques offers a wealth of data that serve as input variables for control systems; even so, it has not yet been figured out how to combine and validate these data in real time with conceptual predicted results or machine learning methods. This may point to a future topic of study. In order to determine the proper placement of the hole design in the future while taking necessary dimensions and positional constraints into account, it is also necessary to develop a novel approach that might predict drilling-caused burr formation in FRPs. As distinct FRP structural composites exhibit varied vibration transmittance while drilling, dynamic design may be used to modify the vibration transmission path inside the composites. The aviation sector is increasingly interested in the use of hybrid-conventional machining methods like ultrasonic-assisted drilling because of its potential to reduce the cutting pressures and wear that are wholly accountable for delaminations. However, the studies related to process mechanisms while drilling of FRP composites using ultrasonic assisted drilling is less [181]. Hence the future research needs to be concentrated more on these hybrid machining techniques to get better quality products.

## 8 Conclusions

The drilled hole quality exhibited by FRP subcomponents is very important for efficient joints in assembly structures. In another direction, the required drilling performance was affected by raised defects such as delaminations, fiber pull-outs, uncut fibers, tool wear, fiber breakage, and dimensional tolerance. The reduction of all these defects with improved surface quality is possible by selecting suitable material geometry, tool type, drilling conditions, hole dimensions, and it is mandatory to study the reinforcement and resin characteristics. This review presented an overview of FRP composites, a literature report on drilling of FRP composites (2000–2010), and the analysis of different drilling conditions and parameters like thrust force, drill geometry, temperature, speed, and feed. It also includes the post-drilling analysis through delaminations, thermal damage, and surface roughness. Furthermore, the recent developments (2011–2021) in CFRP, GFRP, and NFRP composites were studied with both conventional and nonconventional drilling techniques.

When drilling FRP composites, the thrust force, which was directly influenced by the feed rate, was the main factor that affected the delaminations. In every drilling process, there was a possibility of determining the “critical thrust force”, where there was no damage and delaminations below this critical value. More thrust forces are produced as a result of tool wear brought on the continual drilling of holes. The specific drill point angle is one of the main criteria while selecting a drill geometry, and sometimes it leads to more thrust force, which is not acceptable for the drilling process. To achieve a better surface finish with lower thrust forces, the step drill was the most commonly used tool for FRP composites. The chisel edge gives 40–60% of the contribution to the overall generated thrust force. The woven fabric/fiber-reinforced multilayered composites were highly sensitive to damage that occurred through delaminations due to their lower ILSS value. Hence, the drilling tool having a smaller chisel edge was preferred to reduce the thrust force that causes delaminations. Through the rough and fuzzy cuts, the heat produced by the drilling bit reduces the matrix’s stability and results in thermal degradation. The cutting temperature, but at the other hand, causes the matrix to soften and decreases the cutting forces. The main drilling factors that had a big impact on the hole’s surface integrity and drilling temperature were feeds and speed. Cutting speed has a greater impact on surface roughness than feed rate.

The drilling of FRP composites under cryogenic conditions led to a reduction of wall surface roughness and delaminations. However, it generates more thrust forces. When compared to dry drilling, the cryogenic drilling method yields improved dimensional accuracy and allows for faster work rates. This process has a smooth cutting action and reduces tool wear. Among different kinds of geometries, the modified geometry with HSS, tungsten, and solid carbide tools was preferred in most of the experimental research work due to the better surface finish in the drilled holes. In NFRCs, the production of delamination-free holes can be achieved in two ways: either through the chemical treatment of natural fibers through which the bonding is enhanced, or through the drilling of NFRCs with special tool bits. Nonconventional techniques were utilized in much of the research work to get a better hole surface quality and reduce the delaminations and other defects raised during the drilling process. The cost of drilling was high as compared to the conventional drilling process. However, these techniques fulfill the current industry requirements. FRP composite drilling, abrasive water jet machining, laser drilling, and ultrasonic drilling processes were commonly employed for FRP composite drilling. In future, there are many possible trend setting works needs to be highlighted such as self-sharpening drilling tools, recycling of waste fiber chips, and intelligent in-process control system. Moreover, the detailed study of temperature distribution over drilled composite need to analyzed in-depth in concern towards material safety and strength.

## Declarations

### Author contribution statement

All authors listed have significantly contributed to the development and the writing of this article.

## Funding statement

This research did not receive any specific grant from funding agencies in the public, commercial, or not-for-profit sectors.

## Data availability statement

No data was used for the research described in the article.

## Code availability

Not applicable.

## Ethics approval

The authors hereby state that the present work is in compliance with the ethical standards.

## Consent to participate

Not applicable.

## Consent for publication

All authors have read and agreed to publish the manuscript.

## Additional information

The paper’s Corresponding Author, Sanjay Mavinkere Rangappa, works as an Associate Editor at Heliyon Materials Science.

## Declaration of Competing Interest

The authors declare no competing interests.
